# Combining food web and species distribution models for improved community projections

**DOI:** 10.1002/ece3.843

**Published:** 2013-10-21

**Authors:** Loïc Pellissier, Rudolf P Rohr, Charlotte Ndiribe, Jean-Nicolas Pradervand, Nicolas Salamin, Antoine Guisan, Mary Wisz

**Affiliations:** 1 Department of Bioscience, The Arctic Research Centre, Aarhus UniversityNy Munkegade 114, DK-8000, Aarhus, Denmark; 2 Integrative Ecology Group, Estación Biológica de Doñana (EBD – CSIC)Calle Américo Vespucio s/n, Sevilla, 41092, Spain; 3 Department of Ecology and Evolution, University of LausanneBâtiment Biophore, Lausanne, CH-1015, Switzerland

**Keywords:** Biotic interactions, ecological niche modeling, phylogeny, plant–herbivore interactions, trophic network

## Abstract

The ability to model biodiversity patterns is of prime importance in this era of severe environmental crisis. Species assemblage along environmental gradients is subject to the interplay of biotic interactions in complement to abiotic filtering and stochastic forces. Accounting for complex biotic interactions for a wide array of species remains so far challenging. Here, we propose using food web models that can infer the potential interaction links between species as a constraint in species distribution models. Using a plant–herbivore (butterfly) interaction dataset, we demonstrate that this combined approach is able to improve species distribution and community forecasts. The trophic interaction network between butterfly larvae and host plant was phylogenetically structured and driven by host plant nitrogen content allowing forecasting the food web model to unknown interactions links. This combined approach is very useful in rendering models of more generalist species that have multiple potential interaction links, where gap in the literature may occur. Our combined approach points toward a promising direction for modeling the spatial variation in entire species interaction networks.

## Introduction

Sound predictions of the composition and function of future ecosystems are needed to inform decision-making in the face of global change but remain one of the greatest challenges facing ecological scientists (Mokany and Ferrier 2011; Nogués-Bravo and Rahbek 2011). Predicting which species will occur when and where, and their potential interactions requires an understanding of the complex network of trophic linkages that vary in space and time, as well as the associated competitive and facilitative effects (Kissling et al. 2012; Wisz et al. 2013). Species distribution models are spatially explicit models that are used to fill the gaps in our knowledge of spatial distributions of biodiversity, and recent advances in these are used to generate community-level forecasts. These models have only recently begun to incorporate the effect of biotic interactions, but so far, only account for these effects indirectly (e.g., based on correlations in occurrence patterns, Kissling et al. 2012) and cannot divulge information about the way species may or may not interact (Guisan and Thuiller 2005). In contrast to these modeling tools, food web models are used to understand and predict interaction links between species within an interaction network in a given location, as well as measure patterns in the structure of food webs (Naisbit et al. 2012), but these models are generally not spatially explicit (but see Massol et al. 2011). Combining food web models with species distribution models to predict spatial variation in community composition remains an unexplored area in biodiversity modeling but would be a major advancement for predictive ecology.

A common approach to improve species distribution and community models is to consider the distribution of interacting species as a predictor in statistical models (Wisz et al. 2013). This approach is particularly straightforward when examining pairs of species that are known to interact, for example, specialist species when there are at most only a few obligate interactions (e.g., Araújo and Luoto 2007). In such cases, considering positive and negative correlations between species occurrence patterns in models of species distribution and communities generally improves model accuracy (Pellissier et al. 2010; Boulangeat et al. 2012; Giannini et al. 2013; Le Roux et al. 2013). Accounting for correlations in the distributions between species within distribution models can generate useful information on the potential distribution of species given the presence/absence of the known interacting species and has enabled improved forecast of species' fate under future climate change (Schweiger et al. 2012; Giannini et al. 2013). However, this approach is more difficult to apply to more generalist species because it may be unknown how these species interact with others, especially in cases where they do not yet co-occur but may do with global change (Wisz et al. 2013). The difficulty in accounting for species interactions is further heightened when the goal is to model all species in a region and predict community composition or richness. In this case, species distribution modeling lacks the capacity to predict occurrences for all the species in a region, while accounting for multiple and often partially known interactions (Wisz et al. 2013).

Recently, a new approach for modeling food webs was proposed by Rohr et al. (2010). This approach is based on a statistical model that relates the presence and absence of known trophic interactions (links) between species to measureable traits that relate to trophic relationships (such as energy content, size, digestibility, morphological characteristics linked with feeding, etc.) and latent traits (Naisbit et al. 2012). These latent traits are surrogates for properties of interacting species derived from the combined information in the measured traits and are estimated *a posteriori* from the matrix of trophic interactions. The food web model can be interpolated by fitting and predicting linkages between species to the original dataset (here referred as “interpolation”) or extrapolated by projecting the model on to an independent dataset to infer potential links between poorly known species, or species in a different or hypothetical community (Clauset et al. 2008). The capacity to extrapolate a food web model has so far never been exploited, nor have food web models been coupled with species distribution models to predict assemblage structure and function in space and time (Thuiller et al. 2013).

Here, we propose combining the strength of food web models and species distribution models to account for multiple interactions between species. This approach aims to improve community projections by filling in gaps in our knowledge about food webs through accounting for multiple and potentially unknown interaction links across species. Biotic interactions in species distribution models among species of higher or lower trophic positions are quite under-represented (Van der Putten et al. 2010) compared to within trophic level (e.g., Ovaskainen et al. 2010; Laughlin et al. 2012). Extrapolation of food web models within a species distribution modeling framework is therefore a promising approach to fill gaps in our knowledge about the interactions between species and to produce more ecologically realistic forecasts. We test our approach on a comprehensive spatial dataset of plant and herbivore communities distributed along broad environmental gradients in the Swiss Alps (Pellissier et al. 2013).

Because phylogenies provide relevant information to understand and predict species trophic associations and assemblages (Mouquet et al. 2012; Whitfeld et al. 2012; Best et al. 2013; Pellissier et al. 2013), we reconstructed two species-level phylogenies for plant species and butterfly species. We also measured several commonly used plant palatability related traits (specific leaf area, leaf dry matter content, leaf carbon, and nitrogen content Wright et al. 2004) that are used to fit the food web models. Leaf palatability traits represent important components mediating plant–herbivore interactions (Ibanez et al. 2013). We then compared the accuracy of species distribution and community models using only abiotic predictors and considering as a predictor the links inferred from the food web models. We use this approach to address the questions (1) Can statistical food web models be used for extrapolation to an independent community? (2) Does accounting for inferred interaction links in species distribution models improve the accuracy of species distribution and community composition projections?

## Methods

### General framework

**Figure 1:**
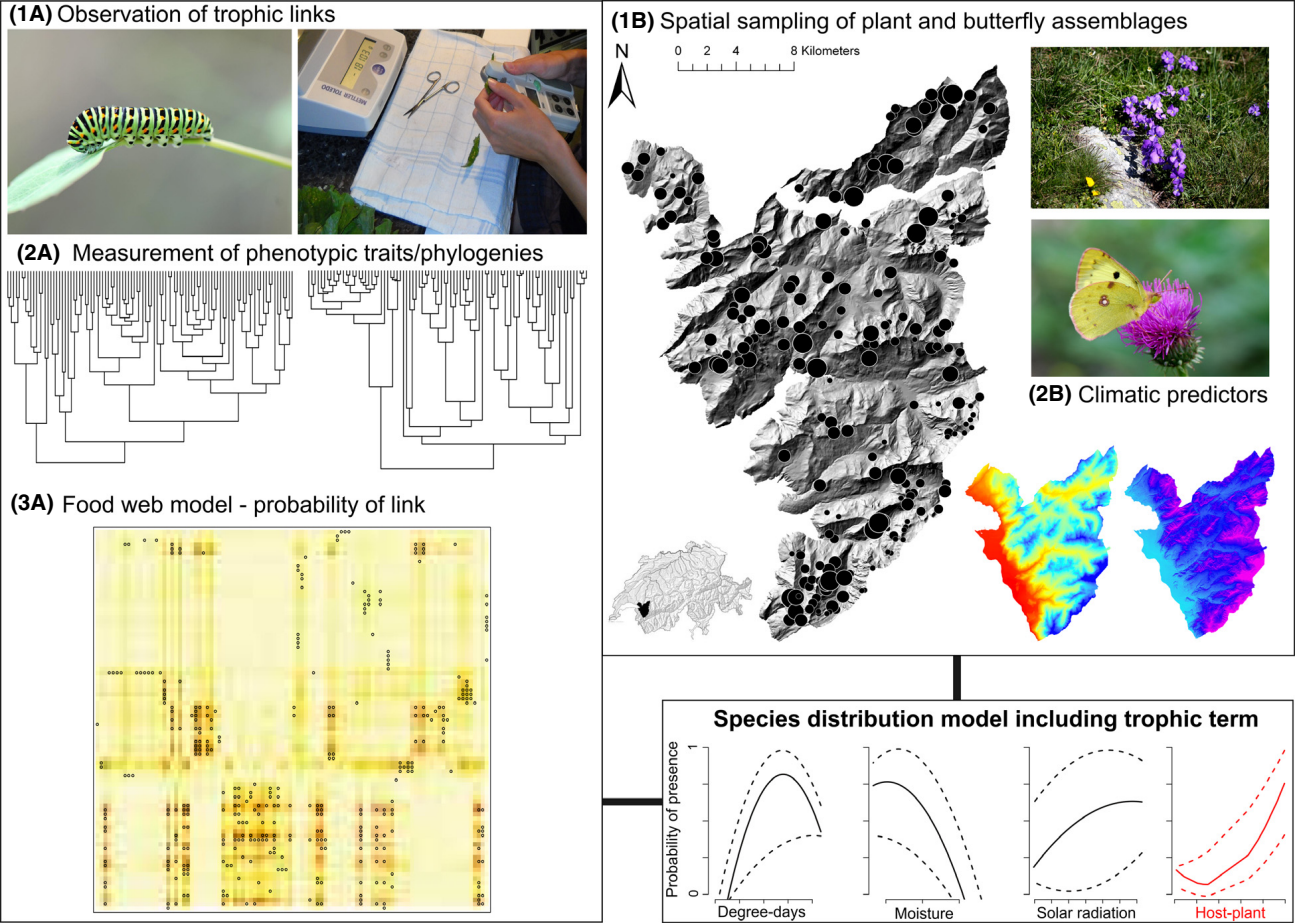
Schema of the methodology and data needed to combine food web and species distribution models. We propose fitting a food web model on observations of trophic interactions between plant and herbivores (1A) in conjunction with traits/phylogenies measured for plant and insects (2A). The food web model provides a probability of link between each pair of plant and herbivore (3A). In parallel, spatial sampling of plant and herbivores (1B, size of the dots proportional to butterfly species richness) allows collecting presence and absence of individual species. The species distribution model finally consists of relating presence and absence of a given herbivore species to climatic predictors (2B), but including a trophic term consisting in the maximal link probability with the plant species at a given site.

The core of our methodology resides in combining statistical models for inferring a plant–butterfly food web and spatial distribution models for projecting the spatial distributions of butterflies. Food webs are only partially known, as observations of herbivores feeding on plants are likely to be incomplete. Furthermore, in the illustration dataset (see description below), 38% of the plant–butterfly pairs of species are never detected in the same place, and for these pairs, the assessment of the presence or absence of a trophic link from field observation is impossible. The inference of the linking probabilities was achieved in two steps. First, we inferred the food web consisting of a species subset with at least one trophic link recorded. We used a statistical model based on latent traits (Rohr et al. 2010) to infer the probabilities of trophic links between plant and butterfly pairs. The model used measurable plant traits as predictors and latent traits to increase the prediction ability. Latent traits are surrogates for properties of interacting species (relating to e.g., foraging sucsceptibility, palatability, or nutrition content) derived from the combined information in the measured traits and are estimated *a posteriori* from the matrix of trophic interactions. The probability of occurrence of species in the landscape was calculated so that they were constrained by their relationship to the abiotic environment, and also the “best-case scenario” trophic link probabilities of the butterfly and plant species present at a given place. This methodology is presented in Fig. 1.

### Food web model

The aim of our statistical food web model is to infer the probability of a trophic link between pairs of butterfly–plant species. This inference is based on the know matrix of trophic interaction *a*_*ij*_, where *a*_*ij*_ = *1* if butterfly species *j* (as a caterpillar) feeds plant species *i*. With such binary data, the standard statistical approach is a logistic regression (also called generalized linear model with binomial distribution) and we model the probability 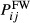 of a trophic links between species *i* and *j*. As a probability takes value between zero and one, the standard approach is to write the model for the logit of the probability. We model this probability using a similar function as in Rohr et al. (2010):


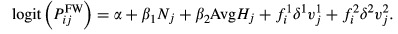
(1)

This model has two parts: (1) a standard linear part, where *N*_*j*_ and *AvH*_*j*_ denote the nitrogen level and average height of plant species *i* (β_1_ and β_2_ are the corresponding slopes, and α is the intercept) and (2) a latent trait part ( 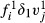 and 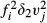 are the corresponding parameter estimates) that quantifies, at the species level, what cannot be captured by the linear part. Each species is characterized by two latent traits, 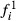 and 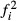 for the butterfly species *j*, and 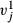 and 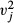 for plants species *i*, and δ_1_ and δ_2_ are scaling factors. Leaves with higher nitrogen content are more palatable and nutritious to herbivores, and larger conspicuous plants may potentially be more susceptible to attract herbivores (Pellissier et al. [Bibr b32][Bibr b33]). Consequently, the two latent traits of each butterfly species can be viewed as species-specific foraging traits, while the plant latent traits can be seen as species-specific susceptibility traits. These latent traits aimed to quantified nonmeasured species' characteristics that are important in explaining the presence or absence of trophic link, while taking into account the measured species' traits (here, the nitrogen level and the average height of plant species). For the latent traits, as the product of the plant vulnerability latent traits and butterfly foraging trait increases, so does the probability that the butterfly feeds on the plant during its caterpillar stage. The value of the latent traits is not know a priori, and they are parameters to be inferred in the food web model from data on observed trophic interactions.

The likelihood function of our latent trait model is given by:


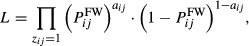
(2)

where, as the trophic interactions are only partially observed, the product only takes into account pairs of species co-occurring at least once, that is, *z*_*ij*_ = *1*. We calculated the number of co-occurrence using the formula presented in Pellissier et al. (2010). We fit the parameters of the model using a Monte Carlo Markov Chain procedure (specifically the Metropolis–Hasting algorithm), adapted from Rohr et al. (2010), equation (2) as the likelihood function. The advantage of using the likelihood function (2) is all the parameters, and the latent traits for all species can be inferred, even if the trophic network is incompletely known. Based on the inferred values, and using the equation (1), we can predict the probability of interaction between all pairs of butterfly and host plant species in the food web.

### Phylogeny as predictor for latent traits

Our observations used to compute values of latent traits come from phylogenetically related species and cannot be considered as independent observations. To account for phylogenetic pseudoreplications, following Felsenstein (1985), we used the phylogenetic correlation to predict the latent traits and also to estimate the trophic link probabilities for all pairs of species.

Here, we first examined whether there was such correlation in the latent traits, using a phylogenetic regression (Freckleton et al. 2002). Such correlation is expected, as plant–herbivore interactions display generally a strong phylogenetic component (Pellissier et al. 2013). A phylogenetic regression is similar to a standard linear regression, only that it does not assume independence of data points, but rather that they are correlated (Freckleton et al. 2002), that is,


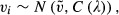


where the variance–covariance matrix C(λ) is given by:


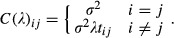


Here, *σ* denotes the variance. This variance–covariance matrix derives the correlation between two latent traits given by:


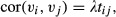


where *λ*, called Pagel's *λ,*is the parameter that controls for the strength in the correlation from the phylogeny and *t*_*ij*_ is the fraction of time common between species *i* and *j* on the phylogenetic tree. When the parameters of the phylogenetic regression are fitted, they can be used to predict the latent traits of a new species using conditional expectation of a normal distribution:


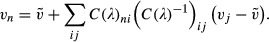
(3)

where the sum is made over all species for which at least one interaction was recorded. Using equation (3), the latent traits values are predicted for all the species with unknown information. Based on these predicted latent traits, the linking probabilities between all pairs of plant–butterfly species are estimated using equation (1). The phylogenetic regression is fitted in R (R Development Core Team 2013), using the library ape. We tested the predictive power of the food web model both internally (fit) and externally using cross-validation. We measured it with the area under the curve (AUC) of a receiver operating characteristic plot (ROC). We tested whether the ability of the food web model to predict butterfly larvae association along with host plants differed among the main butterfly families using a Kruskal–Wallis test.

### Species distribution modeling

We modeled the species distributions using the presence and absence for each species with four different techniques: generalized linear model (GLM), generalized additive model (GAM), gradient boosted model (GBM), and a random forest model (RF). These four statistical techniques are among the most commonly used in species distribution modeling. To produce predictions independent of the calibration data, we ran a tenfold cross-validation. We randomly split the dataset into ten partitions, successively calibrating the models with 90% of the data and sequentially predicting the species distributions and communities based on the remaining 10% data. We used three steps to model the distribution of each species. First, we modeled the distribution of the species using only abiotic predictors.

For the GLM model, the probability of presence of a given butterfly species at a site k is then given by


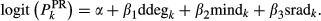


where ddeg_*k*_ denotes the degree-days, mind_*k*_ the moisture, and srad_*k*_ the solar radiations. Second, in concert with the abiotic predictors, we added a biotic predictor comprising the presence or absence at a given site of the obligatory trophic partner from the biotic interactions as provided by the literature. Third, alongside the abiotic predictors, we added a biotic predictor comprising the highest probability of an interaction link with any of the plants occurring at the site given by the food web model. For the GLM model, the probability of presence of a given butterfly species at site k is then given by


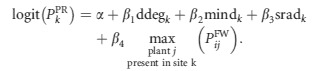


where 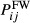 is the maximum or “best-case scenario” trophic link probability predicted by our food web model.

We calculated the predictive power of the SDMs for each species, technique and set of predictors using the AUC. In addition, to obtain the community at each site, we stacked all the species predicted using the threshold independent resampling approach developed in Dubuis et al. (2011) and Pellissier et al. (2013). We evaluated the projected community composition to observed values using the Sørenson index and compared the accuracy between abiotic and biotic SDMs. The Sørenson index is computed as follow:


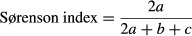


where *a* is the number of species that are both observed and predicted as being present, *b* is the number of species observed as present but predicted as absent, and *c* is the number of species observed as absent but predicted as present. Finally, we also compared species richness between observed and predicted values following Pellissier et al. (2012a,b) to assess whether richness predictions are more accurate, once trophic interactions are included in the models. Species distribution models were run in R using the libraries referred in Thuiller et al. (2009). We compared the species distribution and communities composition predictive accuracies between abiotic and biotic models using paired Wilcoxon signed-rank tests.

### Species data

To test our framework, we used a dataset collected in the Western Swiss Alps spanning an elevation gradient from 1000 m to 3210 m a.s.l. (Fig. 1, Pellissier et al. 2013). Site selection, which only included open vegetation grasslands, was conducted following a balanced stratified random sampling design based on elevation, slope, and aspect in open grasslands (Hirzel and Guisan 2002). Sampling in forests was avoided because they constitute suboptimal habitats for the majority of Papilionoidea species in the study area. Between May and September 2009 and 2010, 192 plots of 50 × 50 m were sampled across the whole study area (700 km^2^, Fig. S1). All butterfly species belonging to the Papilionoidea super-family (sensu Heikkilä et al. 2012) were monitored. We collected adult specimens instead of caterpillars because they are more conspicuous, more reliable to survey and easier to identify. For modeling purposes, we only considered species with at least 10 occurrences and excluded very mobile species, as these are not necessarily related to environments or resources where they occur and may constitute noise in our analyses (77 species in total). Because occurrences of the butterfly species from our dataset had a low degree of spatial autocorrelation (mean Moran's I = 0.14, range −0.04 to 0.55), we can be confident about the statistical independence of the inventoried sites (see also Randin et al. 2009). Plant species as potential hosts for butterfly larvae were exhaustively inventoried in 4 m^2^ subplots at the center of each butterfly plot. We ensured that the vegetation was representative of the entire 2500 m^2^ area from which the butterflies were monitored. For each site, we extracted climatic predictors known to influence plant and insect establishment (Hodkinson 2005). All predictors were interpolated from meteorological stations using a digital elevation model (DEM) at 100 m resolution. Values for degree-days (above 0°C) and moisture index (computed as the difference between precipitation and evapotranspiration) were interpolated following Zimmermann and Kienast (1999). Solar radiation values were calculated using the tool implemented in ArcGIS 10. For each plot location, we extracted the values of environmental variables from the associated environmental layers.

To model the trophic interactions between each butterfly and host plant using the food web model, we collected feeding preferences for the butterfly species recorded in the plots from the literature (Pellissier et al. 2013). In addition, leaf traits were gathered for all the 215 most frequent and abundant vascular plant species occurring in the study area (from a total of ∼700 recorded species) and span major portions of the angiosperm phylogeny (Pellissier et al. 2013). Leaf nitrogen (N) was measured from grounded dried leaves and was analyzed by combustion with an elemental analyser. Canopy height was measured from 10 individuals spanning the species ecological conditions. In addition, the phylogenetic relationships of all butterfly species found in the study area, and the 215 plant species were inferred using DNA sequences following methods described in Pellissier et al. (2013) (see also Supplementary materials, Figures S1 and S2).

## Results

### Plant traits and phylogeny predict butterfly and host plant interactions

**Figure 2:**
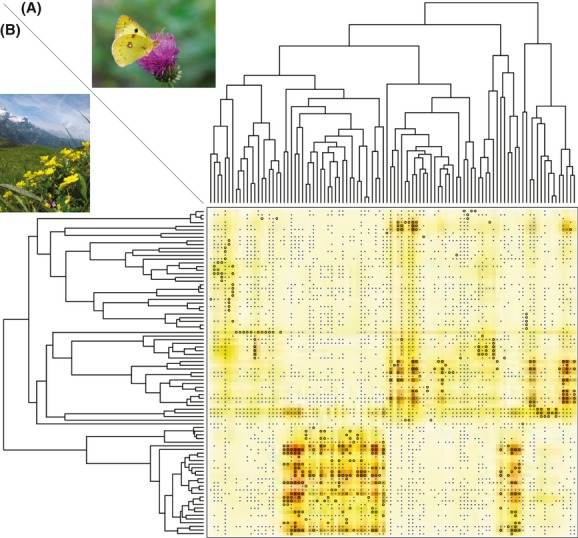
Trophic interactions matrix between butterfly species (A) and plant species (B). Each column and row represents a butterfly and a plant species, respectively. A black dot at an intersection represents a trophic interaction between the two corresponding species, while a blue dot indicates the absence of co-occurrence. Plants and butterfly phylogenies are presented on the left and bottom of the trophic interaction matrix. Phylogenetically closely related species tend to interact with the same subset of species. Our statistical food web model uses the trophic information on co-occurring species to infer the probability of a trophic link between all pair of species. The magnitudes of these probabilities are represented by the background color, increasing from pale yellow to red.

We fitted the food web model on 89 plant species that are known to be eaten by at least one butterfly species (Fig. 2). Based on this calibration dataset, the food web model was able to predict the trophic associations between butterfly species and their respective host plants both internally (AUC fit = 0.96) and externally (AUC cross-validation = 0.84). We found a significant effect of plant nitrogen content in the susceptibility of being a trophic resource for butterfly larvae (slope = 0.145, *P* = 0.002). In addition, we found that the two latent traits were significantly correlated with the plant and butterfly phylogenies. Based on the phylogenetic regression using Pagel's-λ correlation structure, we found for plants latent traits 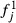: Pagel's-λ = 0.8217 *P*-value < 0.001, plants latent traits 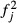: Pagel's−λ = 0.6497 *P*-value < 0.001, and for butterfly latent traits 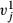: Pagel's−λ = 0.7257 *P*-value < 0.001, butterfly latent traits 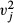: Pagel's−λ = 0.9230 *P*-value < 0.001. The ability of the food web model to predict butterfly larvae association along with host plants differed among the main butterfly families (Kruskal–Wallis test, KW = 17.06, *P* = 0.004). The highest predictive performance was achieved for the families Nymphalidae and Pieridae, followed by Hesperiidae, while the weakest performance was achieved for Lycaenidae, Papapilionidae, and the Riodinidae species (*Haemearis lucina*). (Fig. S3).

### Species distribution

Accounting for modeled trophic links between each butterfly and host plant in models of species distributions significantly improved the predictive power of the models (measured using AUC) compared with a model with only abiotic components (paired Wilcoxon test, GLM: *V* = 790, *P* = 0.01, GAM: *V* = 858, *P* = 0.03, GBM: *V* = 843, *P* = 0.03, RF: *V* = 758, *P* = 0.008, Fig. S4). Model accuracy for some species was largely improved by accounting for the potential trophic links with host plants (e.g., *Parnassius appolo* +21%, *Pontia callidice* +15%, *Papilio machaon* +10%). In contrast, the predictive power of SDMs for some species declined by incorporating the biotic component (e.g., *Pyrgus andromedae* −9%, *Polyommatus eros* −7%, *Plebejus argus* −4%).

We found no general improvement in predictive power when species interactions included in the models were directly based on the literature (paired Wilcoxon test, GLM: *V* = 1150, *P* = 0.55, GAM: *V* = 1167, *P* = 0.57, GBM: *V* = 1289, *P* = 0.82, RF: *V* = 1129, *P* = 0.47) or when the food web was only interpolated to the 89 plant species used for calibration and those used in the SDMs (paired Wilcoxon test, GLM: *V* = 1050, *P* = 0.48, GAM: *V* = 1172, *P* = 0.59, GBM: *V* = 1340, *P* = 0.84, RF: *V* = 1127, *P* = 0.47).

Only models for species from the Nymphalidae (paired Wilcoxon test, GLM: *V* = 161, *P* = 0.04, GAM: *V* = 166, *P* = 0.04, GBM: *V* = 149, *P* = 0.03, RF: *V* = 139, *P* = 0.01) and Pieridae (paired Wilcoxon test, GAM: *V* = 2, *P* = 0.04, GBM: *V* = 2, *P* = 0.04, RF: *V* = 2, *P* = 0.01) families were improved using the food web model compared with the model using the raw literature data. Hesperiidae (paired Wilcoxon test, GLM: *V* = 44, *P* = 0.85, GAM: *V* = 46, *P* = 0.87, GBM: *V* = 31, *P* = 0.45, RF: *V* = 25, *P* = 0.25) and Lycaenidae (paired Wilcoxon test, GLM: *V* = 72, *P* = 0.56, GAM: *V* = 69, *P* = 0.53, GBM: *V* = 70, *P* = 0.55, RF: *V* = 69, *P* = 0.53) showed no significant difference in predictive power.

### Species richness and communities composition

**Figure 3:**
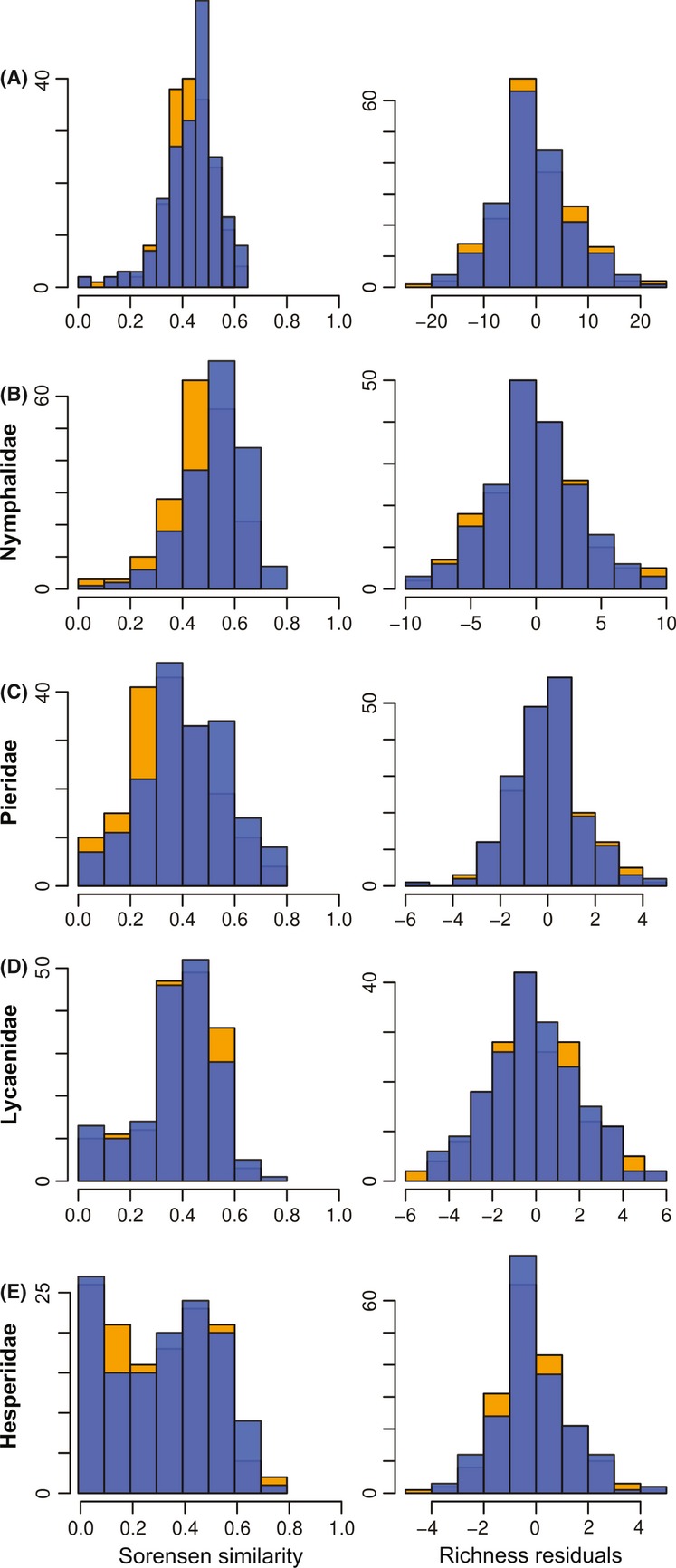
Histograms of Sørenson similarity with observations and richness residuals of the stacked species distribution models. Shown are the results for the random forest modeling technique with abiotic predictors (in orange below) and considering in addition the trophic links with host plant (in blue), for all species together (A) and the four main families, Nymphalidae (B), Pieridae (C), Lycaenidae (D), and Hesperiidae (E).

Community predictions were also improved with a relative increase of 11% in the median composition similarity (paired Wilcoxon test on Sørenson index: GLM: *V* = 6950, *P* = 0.008, GAM: *V* = 6867, *P* = 0.006, GBM: *V* = 7230, *P* = 0.02, RF: *V* = 7157, *P* = 0.01, Fig. 3). Across the communities, we found a Sørenson index of 0.46 between observed and predicted species when trophic interactions were accounted for in the models, while 0.41 when interactions were not accounted for. In addition, we found a 7% decrease in the median of absolute values of modeled richness residuals (paired Wilcoxon test on richness residuals: GLM: *V* = 10350, *P* = 0.007, GAM: *V* = 10420, *P* = 0.004, GBM: *V* = 9967, *P* = 0.04; RF: *V* = 9779, *P* = 0.05).

## Discussion

In nature, species' interactions and interdependencies are dynamic under the processes of global change, and ecological constraints within food web are among those biotic interactions demonstrated to affect species' spatial distributions (Van der Putten et al. 2010; Wisz et al. 2013). Although there have been recent advances in predicting the distributions of species that potentially contribute to community function (Thuiller et al. 2013), it is still a challenge to predict the identity of the species that will interact with each other across trophic levels at a given place or time (Mokany and Ferrier 2011; Mokany et al. 2011). This study presents a novel approach that addresses this challenge by combining the strengths of food web and species distribution modeling (SDMs) to produce more ecologically meaningful predictions of species distribution and assemblage composition. As such, it can be applied to any ecosystem where occurrence data and environmental predictors are available for generating species distribution predictions, and where a general food web model can be constructed based on quantifiable species' traits and phylogenetic information complementing a partial food web. Here, we illustrate the utility of this combined approach, examining 125 butterfly species and 215 potentially interacting plant species in the western Swiss Alps. By combining a food web model based on plant resource characteristics, or phylogenetic information, and SDMs, we show that accounting for trophic interactions between a butterfly and associated food plant host improves the quality of community forecasts.

### Extrapolation of food webs

Given the prevalence of functionally constrained food web networks in several ecosystems across the globe (e.g., Trussell et al. 2002; Petchey et al. 2005), our approach shows great promise for providing more ecologically informed and realistic spatial distributions for species including those that have incomplete distributional information or when it is unknown how they interact with other species. A limited number of studies have attempted to integrate trophic interactions in biogeographical modeling, including Gravel et al. (2011), who extended the island biogeography theory of extinction-colonization processes to account for trophic interactions. Similarly, Jabot and Bascompte (2012) integrated a spatial meta-community structure to food web network approaches by studying a bitrophic plant–pollinator interaction. This is the first attempt to combine a food web model with a niche-based species distribution model. The ability to extrapolate food web models is useful in the context of climate change, to predict potential interactions of species that do not currently co-occur or interact, but may do so in the future. For example, a recent study documented a temperature-dependent shift of a butterfly species to a novel host plant impacting species range (Pateman et al. 2012; but see Pelini et al. 2010). Our food web model SDM approach could be used to predict shifts such as this one.

A unique feature of our approach is the extrapolation of the food web model to predict missing interactions in networks and inform species distribution models. Our food web modeling approach yields a continuous index of interaction probability, based on the quantitative support for the presence or absence of interactions between species derived from information on species' traits and phylogeny. Information directly derived from the literature is less reliable, as there is typically underrepresentation of information on the interactions among species. Here, based on known interactions involving 125 butterfly and 89 host plants, we extrapolate a food web model to 215 host plants (∼35% of the regional species pool) for which relevant functional traits and phylogenetic positions were available. The extrapolation was based on the strong associations between nitrogen content and the probability of a trophic interaction between species and the correlation between the latent traits and the species phylogeny. This supports the view that herbivore diet shows strong phylogenetic constraints (Pellissier et al. 2013) and is driven by plant resource content (Pellissier et al. 2012a,b). Previous studies also showed that food webs may be phylogenetically structured (Ives and Godfray 2006). We found no general improvement of species distribution models when we considered only interpolated values on the 89 host plants (∼15% of the plant species pool) or using the raw links in the literature. This may have arisen because too many potential host plants were missing resulting in biased inferences of butterfly presence and absences at sites. We speculate that more accurate assemblage predictions may have been realized if we had information for the full regional species pool (∼700 plant species), but for which trait information is currently unavailable.

Our approach is particularly suited for group of species that are neither specialist nor extreme generalists, but still interact with a large number of species, where current knowledge on species interactions is probably incomplete. From our dataset, the models built for the butterfly families Nymphalidae and Pieridae showed the most consistent improvement in model predictive performance (Fig. 3). This may perhaps be due to the Nymphalidae family being composed of more polyphagous species (larger number of potential host plants) and the display of lower phylogenetic variability in host plant association (e.g., Satyrinae subfamily feeding on the grass family, Poaceae and Cyperaceae) (Pellissier et al. 2013). Consequently, the food web model performed relatively well across this family and could explain several missing links in the interaction matrix required to predict communities using SDMs. Models for species of the Lycaenidae and Hesperiidae were not improved when information from the food web model was included in SDMs. Those families display higher degree of shifts in host-plant associations with closely related lineages specialized on distinct plant clades (Megens et al. 2005; Vila et al. 2011). As a consequence, trophic interactions are more difficult to model and including uncertain interaction probabilities results in poorest predictions. The overall trend of improvement at the species and community levels is likely to arise because the more generalist Nymphalidae family is dominant in our butterfly dataset, while specialist species are less frequent (Fig. 3).

One caveat of the approach proposed in this study is that it is bound to a statistical framework and cannot demonstrate causality. Even if species ecological knowledge supports the relevance of including trophic interactions in model of species distribution, adding further abiotic predictors may also have led to model improvement. Hence, we are unable to demonstrate that the inclusion of trophic interactions actually improves the models in a causal and mechanistic way. Nevertheless, despite the fact that our results are correlative, they suggest new research areas related to the inclusion of trophic interactions in studies of species distributions.

### Future perspectives

In addition to improving model predictions, the next-generation models combining SDMs and food web models should allow novel and complex ecological questions to be addressed (Thuiller et al. 2013). For instance, how might recurrent perturbations in the food web (e.g., the extinction of a predator) affect the distribution of species in a landscape? Nevertheless, the approach presented here is a timely contribution in using food web models to infer the geographical distribution of species in the landscape, as recently proposed by Massol et al. (2011) and Jabot and Bascompte (2012). In addition, we considered a food web that only accounts for bottom-up interactions that potentially influence species' distributions (i.e., the host plant resources that simultaneously influence butterfly distribution). Our approach can be extended to both bottom-up and top-down interactions within food webs. However, this would require a statistical model that simultaneously fits both food web structure and the presences/absences of interactions along environmental gradients, while considering the turnover in these interactions in time and space.

A key improvement of our approach is that with widespread species, trophic interactions usually shift across a species range, and as yet cannot be accounted for in SDMs that do not incorporate food web models. With the plant–butterfly system, we have illustrated, as plant leaf palatability (SLA, LDMC) shift along environmental gradients, this may in turn influence the interactions with other species along those gradients (Linder et al. 2012). In such a landscape, food web models can potentially account for shifts in prevailing environmental conditions, an area to be considered in future modeling efforts. As with many interdisciplinary modeling efforts, the refinement of our models is constrained by the availability of data to fit and evaluate them. For instance, the dataset used to test these methods are hindered by a spatial scale mismatch between plant and butterfly taxa. While the butterfly communities were sampled in plots of 50 × 50 m, only 2 × 2 m grasslands were sampled for plant species presences. It is probable that if we had collected host plant data at a scale more representative of the butterfly plots, better results may have been achieved. In addition, because of the difficult detection of larvae in the field, we used presence and absence of adult butterfly specimens, while we modeled the food web of larvae. It is likely that the difference between abiotic models and those considering trophic interactions would have been more pronounced if records of larvae were used.

## Conclusion

Here, we facilitate the integration of ecological rules based on trophic interactions to constrain prediction of species assemblages in space and time. The models built using our combined approach are ecologically more sounds as they consider biotic interaction that are known a priori to influence the life history of butterflies. This framework extends the boundaries of SDMs by accounting for known and unknown species ecological interactions and produces more realistic models of species' assemblages. It also offers an innovative way to improve our understanding of community assembly by accounting for the multiple interactions among different species based on extensive datasets. Moreover, it informs where species can be found as a function of their relation to the abiotic environment, as well as how these species will interact with novel components of the biotic environment. Our approach draws upon the strengths of spatial ecology and trophic ecology, and should encourage the further integration of these disciplines to advance efforts to predict species assemblages.

## Supporting Information

**Figure S1.** Butterfly phylogeny.

**Figure S2.** Molecular dated phylogenetic tree of 231 angiosperm species from the Western Swiss Alps.

**Figure S3.** Trophic interactions matrix between butterfly species and plant species extrapolated on the full dataset comprising 125 butterfly and 215 plant species.

**Figure S4.** Boxplot of the AUC of the modeled links of the food web model for each of the four main butterfly families, Nymphalidae, Pieridae, Lycaenidae, Hesperiidae.

**Figure S5.** Histograms of the difference between the predictive power of the models (AUC) without and with the trophic interaction as a predictor.

**Table S1.** Species belonging to Papilionoidea that were found in the study area.
