# Supplementary material for: Combining food web and species distribution models for improved community projections
Source: Ecol Evol. 2013 Oct 21;3(13):4572–83. doi: 10.1002/ece3.843 (PMC3856755; doi:10.1002/ece3.843)
Supplement: Supplementary file 1 [file ece30003-4572-SD1.docx]

**Supplementary materials**

**Figure S1** – Butterfly phylogeny. Shown is the consensus tree using a Bayesian ultrametric approach of the species of butterfly species found in the study area, belonging to the six families of butterflies, viz. Nymphalidae, Lycaenidae, Pieridae, Papilionidae, Riodinidae and Hesperiidae. Phylogenetic relationships were inferred using DNA sequences obtained from GenBank and including two nuclear markers (EF1-alpha, Wgl) and four mitochondrial markers (16s, COI, NDH1, NDH5).


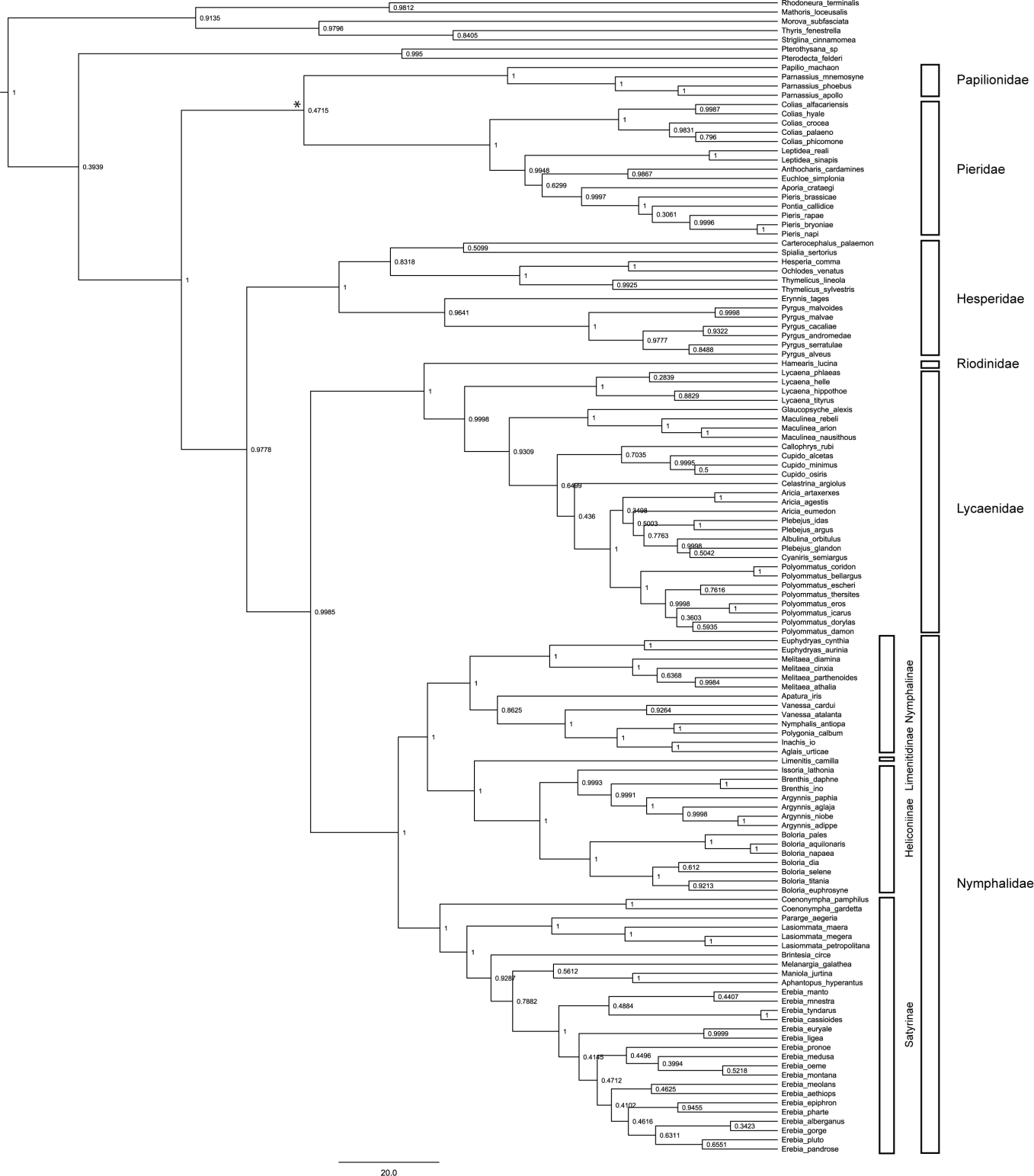


**Figure S2** ˗ Molecular dated phylogenetic tree of 231 angiosperm species from the Western Swiss Alps. Posterior means of divergence are in relative time units with all nodes scaled from a common basal divergence of 130 Mya. The nodes were well resolved and supported, with 77% nodes receiving posterior probabilities greater than 90%. Two outgroup species *Abies alba* Mill. and *Picea abies* (L.) H. Karst. were used to root the tree. The resulting phylogenetic trees were checked against the Angiosperm Phylogeny Group tree for accepted relationships among plant orders and families The phylogenetic reconstructions, including divergence time estimations, produced well-supported phylogenetic trees with nodes congruent to taxonomic groups defined by the APG III classification. A total of 56% of the nodes in the dated tree had 100% bootstrap support. A total of 77% nodes had posterior probabilities greater than 90%. In general, only a few nodes showed low support, such as the placement of *Cirsium spinosissimum* (L.) Scop. and *Cirsium oleraceum* (L.) Scop. within the Asteraceae family, and some nodes within the Cyperaceae and Lamiaceae family.


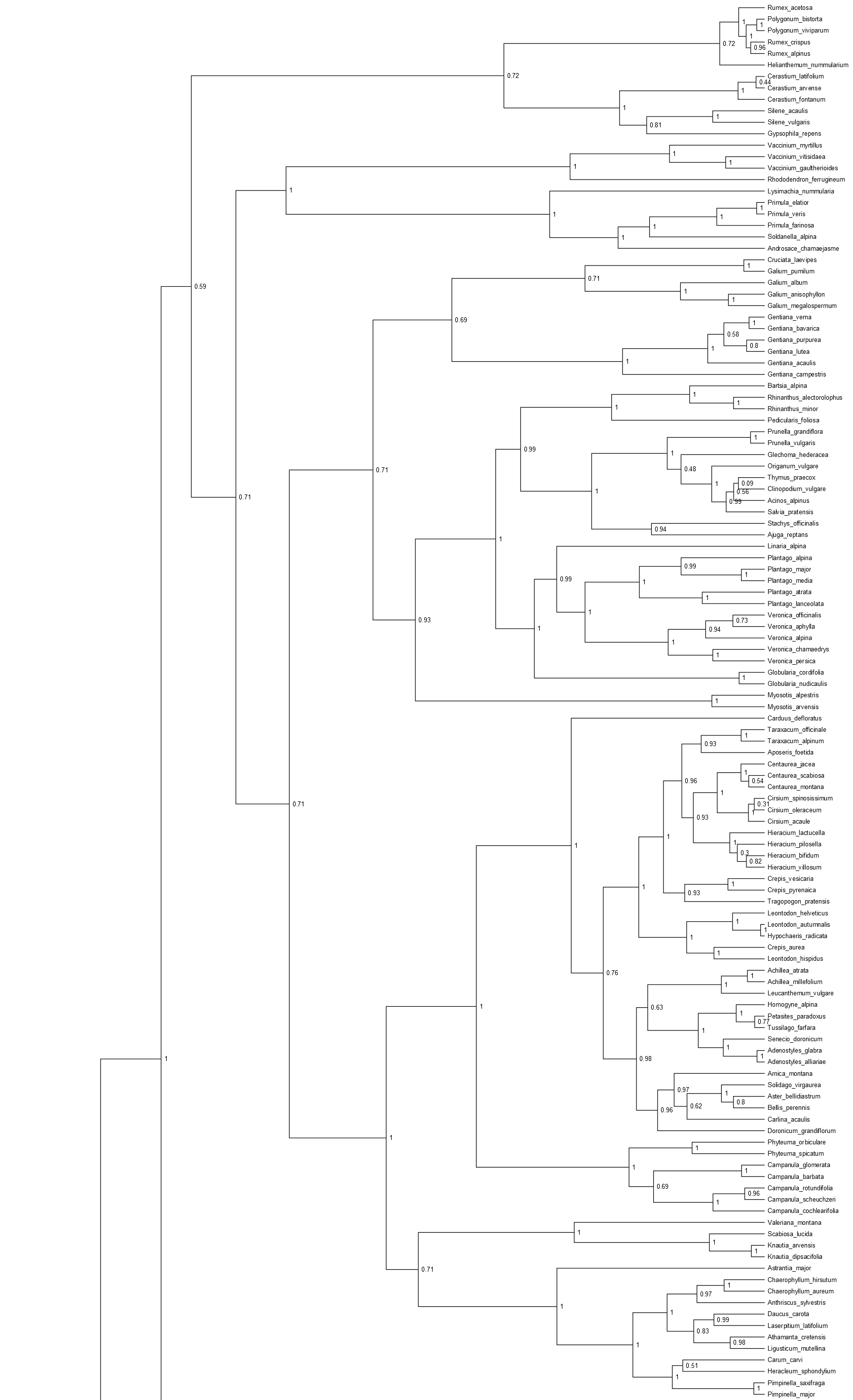


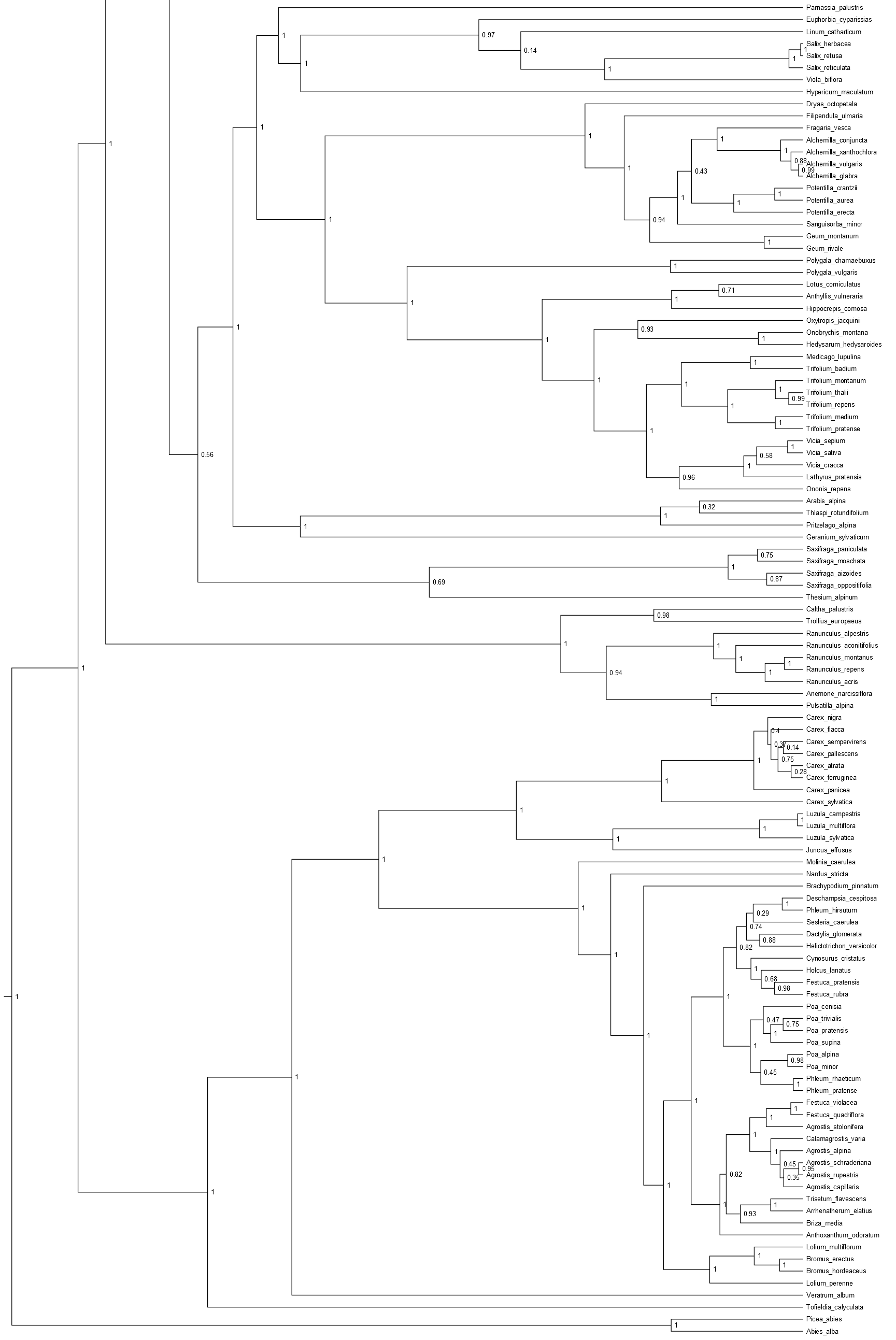


**Figure S3** - **Trophic interactions matrix between butterfly species and plant species extrapolated on the full dataset comprising 125 butterfly and 215 plant species.** Each column and row represents a butterfly and a plant species respectively. A black dot at an intersection represents a trophic interaction between the two corresponding species, while a blue dot indicates the absence of co-occurrence. Plants and butterfly phylogenies are presented on the left and bottom of the trophic interaction matrix.


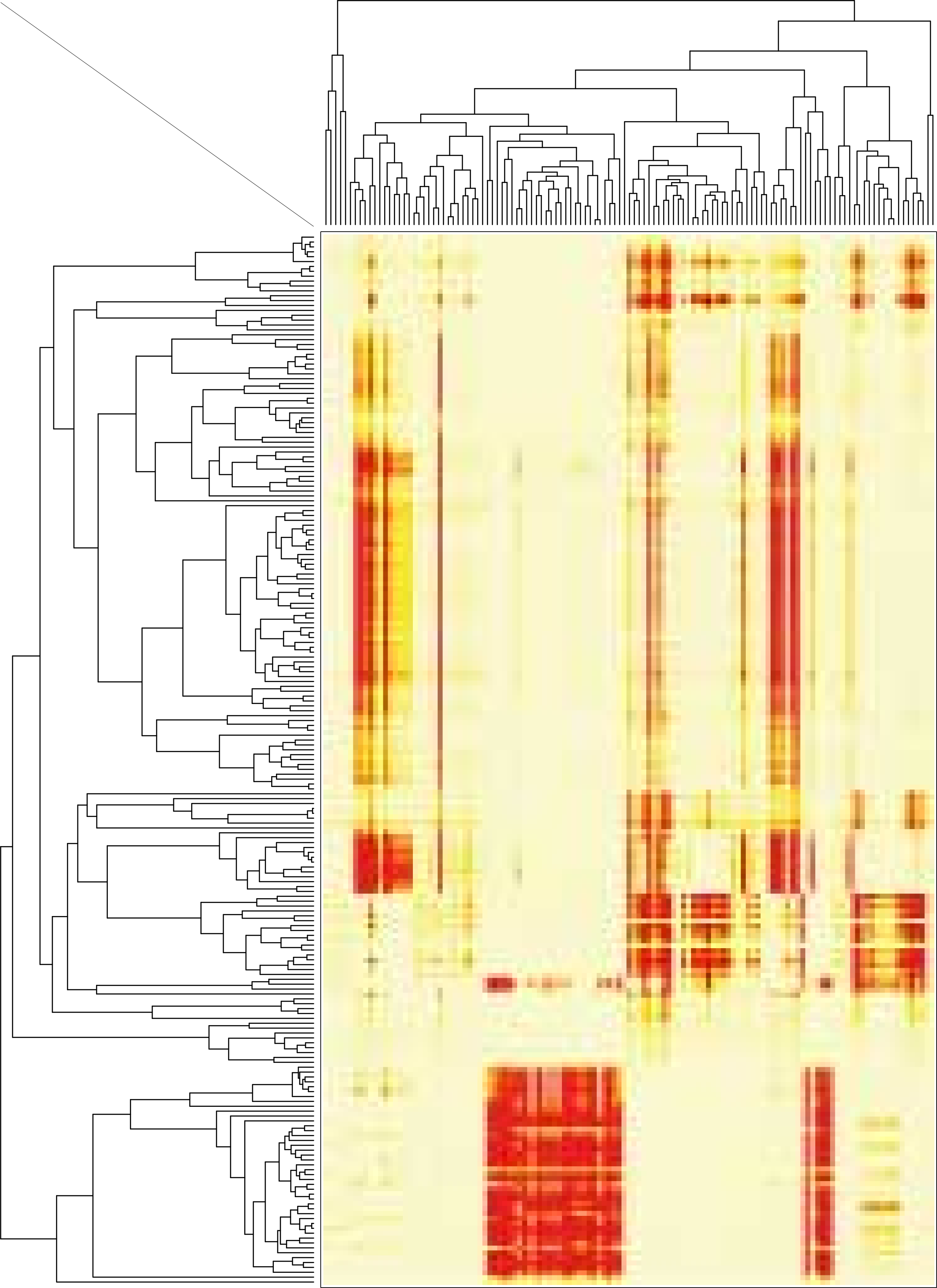


**Figure S4** - Boxplot of the AUC of the modelled links of the food-web model for each of the four main butterfly families, Nymphalidae, Pieridae, Lycaenidae, Hesperiidae. The food web model was more limited in predicting the interactions links of Lycaenidae, while it scored the highest for Nymphalidae and Pieridae. Nevertheless, a few more specialized Nymphalidae species had a lower score especially in the *Boloria*, *Euphydryas* and *Melitea* genera.

**Figure S5** - Histograms of the difference between the predictive power of the models (AUC) without and with the trophic interaction as a predictor. Results for the four modelling techniques (GLM, GAM, GBM and RF) are presented.


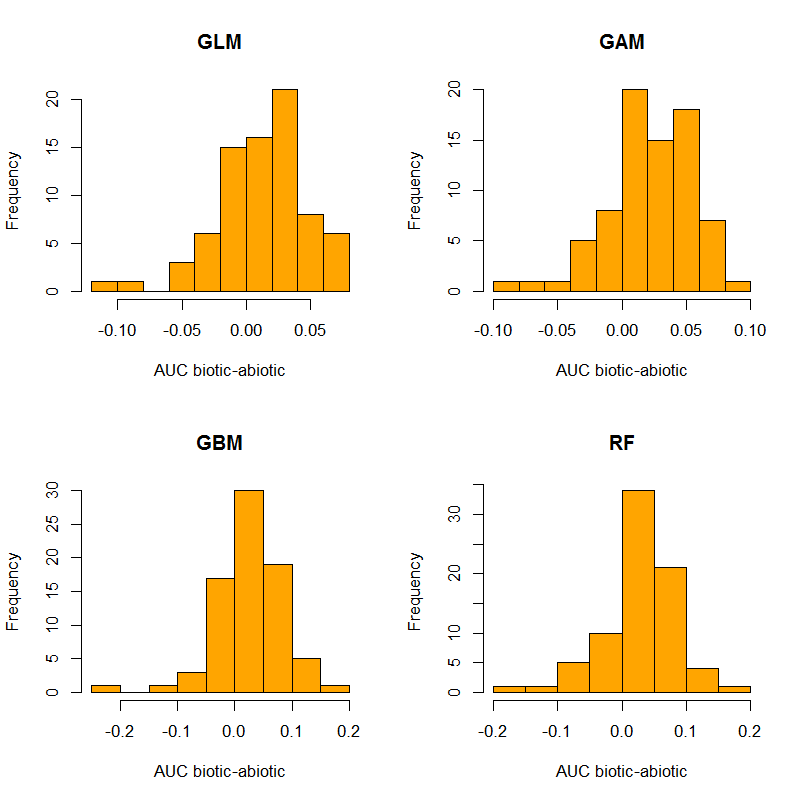


**Table S1** - Species belonging to Papilionoidea that were found in the study area.

| **Species** | **Observations** |
| --- | --- |
|  |  |
| ***Hesperiidae*** |  |
| *Carterocephalus palaemon* | 18 |
| *Erynnis tages* | 57 |
| *Hesperia comma* | 34 |
| *Ochlodes venatus* | 29 |
| *Pyrgus alveus* | 34 |
| *Pyrgus andromedae* | 10 |
| *Pyrgus cacaliae* | 10 |
| *Pyrgus carlinae* | 1 |
| *Pyrgus malvae* | 23 |
| *Pyrgus malvoides* | 4 |
| *Pyrgus serratulae* | 35 |
| *Spialia sertorius* | 12 |
| *Thymelicus lineola* | 72 |
| *Thymelicus sylvestris* | 31 |
|  |  |
| ***Lycaenidae*** |  |
| *Aricia agestis* | 1 |
| *Aricia artaxerxes* | 45 |
| *Aricia eumedon* | 46 |
| *Callophrys rubi* | 28 |
| *Celastrina argiolus* | 2 |
| *Cupido alcetas* | 1 |
| *Cupido minimus* | 131 |
| *Cupido osiris* | 2 |
| *Glaucopsyche alexis* | 1 |
| *Lycaena helle* | 5 |
| *Lycaena hippothoe* | 35 |
| *Lycaena phlaeas* | 2 |
| *Lycaena tityrus* | 29 |
| *Maculinea arion* | 46 |
| *Maculinea nausithous* | 2 |
| *Maculinea rebeli* | 1 |
| *Plebeius glandon* | 18 |
| *Plebeius orbitulus* | 10 |
| *Plebeius argus* | 20 |
| *Plebeius idas* | 7 |
| *Polyommatus damon* | 27 |
| *Polyommatus semiargus* | 101 |
| *Polyommatus bellargus* | 21 |
| *Polyommatus coridon* | 39 |
| *Polyommatus dorylas* | 9 |
| *Polyommatus eros* | 26 |
| *Polyommatus escheri* | 1 |
| *Polyommatus icarus* | 51 |
| *Polyommatus thersites* | 12 |
|  |  |
| ***Riodinidae*** |  |
| *Hamearis lucina* | 11 |
|  |  |
| ***Nymphalidae*** |  |
| *Aglais urticae* | 166 |
| *Apatura iris* | 1 |
| *Argynnis adippe* | 18 |
| *Argynnis aglaja* | 73 |
| *Argynnis niobe* | 26 |
| *Argynnis paphia* | 9 |
| *Boloria dia* | 6 |
| *Boloria euphrosyne* | 52 |
| *Boloria selene* | 2 |
| *Boloria titania* | 76 |
| *Boloria aquilonaris* | 2 |
| *Boloria napaea* | 40 |
| *Boloria pales* | 70 |
| *Brenthis daphne* | 7 |
| *Brenthis ino* | 17 |
| *Euphydryas aurinia* | 60 |
| *Euphydryas cynthias* | 3 |
| *Inachis io* | 9 |
| *Issoria lathonia* | 8 |
| *Limenitis camilla* | 1 |
| *Melitaea athalia* | 36 |
| *Melitaea parthenoides* | 8 |
| *Melitaea cinxia* | 5 |
| *Melitaea diamina* | 62 |
| *Nymphalis antiopa* | 1 |
| *Polygonia c.album* | 6 |
| *Vanessa cardui* | 56 |
| *Vanessa atalanta* | 28 |
| *Aphantopus hyperantus* | 36 |
| *Brintesia circe* | 3 |
| *Coenonympha gardetta* | 77 |
| *Coenonympha pamphilus* | 47 |
| *Erebia aethiops* | 83 |
| *Erebia alberganus* | 9 |
| *Erebia cassioides* | 12 |
| *Erebia epiphron* | 26 |
| *Erebia eriphyle* | 5 |
| *Erebia euryale* | 40 |
| *Erebia gorge* | 22 |
| *Erebia ligea* | 47 |
| *Erebia manto* | 79 |
| *Erebia medusa* | 6 |
| *Erebia melampus* | 87 |
| *Erebia meolans* | 9 |
| *Erebia montana* | 6 |
| *Erebia oeme* | 77 |
| *Erebia pandrose* | 38 |
| *Erebia pharte* | 57 |
| *Erebia pluto* | 18 |
| *Erebia pronoe* | 22 |
| *Erebia tyndarus* | 28 |
| *Erebia mnestra* | 1 |
| *Lasiommata maera* | 60 |
| *Lasiommata megera* | 5 |
| *Lasiommata petropolitana* | 35 |
| *Maniola jurtina* | 44 |
| *Melanargia galathea* | 31 |
| *Oeneis glacialis* | 4 |
| *Pararge aegeria* | 8 |
|  |  |
| ***Papilionidae*** |  |
| *Papilio machaon* | 86 |
| *Parnassius apollo* | 20 |
| *Parnassius mnemosyne* | 7 |
| *Parnassius phoebus* | 6 |
|  |  |
| ***Pieridae*** |  |
| *Anthocharis cardamines* | 46 |
| *Aporia crataegi* | 58 |
| *Colias alfacariensis* | 20 |
| *Colias crocea* | 20 |
| *Colias hyale* | 9 |
| *Colias palaeno* | 6 |
| *Colias phicomone* | 72 |
| *Euchloe simplonia* | 5 |
| *Leptidea reali* | 7 |
| *Leptidea sinapis* | 5 |
| *Pieris brassicae* | 46 |
| *Pieris bryoniae* | 72 |
| *Pieris napi* | 66 |
| *Pieris rapae* | 70 |
| *Pontia callidice* | 12 |
